# Prediction of all-cause death using ^11^C-hydroxyephedrine positron emission tomography in Japanese patients with left ventricular dysfunction

**DOI:** 10.1007/s12149-016-1081-z

**Published:** 2016-05-18

**Authors:** Wataru Fujita, Ichiro Matsunari, Hirofumi Aoki, Stephan G. Nekolla, Kouji Kajinami

**Affiliations:** 1 Department of Cardiology, Kanazawa Medical University, 1-1 Daigaku, Uchinada, Kahoku, Ishikawa 920-0293 Japan; 2 The Medical and Pharmacological Research Center Foundation, Ishikawa, Japan; 3 Division of Nuclear Medicine, Department of Radiology, Saitama Medical University Hospital, 38 Morohongo, Moroyama, Iruma, Saitama 350-0495 Japan; 4 Department of Nuclear Medicine, Technical University Munich, Munich, Germany

**Keywords:** Nervous system, Radioisotopes, Sympathetic, Tomography

## Abstract

**Objectives:**

The aim of this study was to determine whether ^11^C-hydroxyephedrine (^11^C-HED) can predict adverse events including all-cause death in Japanese patients with left ventricular (LV) dysfunction.

**Background:**

Although ^11^C-HED PET has been used to assess cardiac sympathetic innervation in various disease conditions, data on their prognostic value are limited.

**Methods:**

Sixty patients (mean LVEF, 42 ± 14 %) with LV dysfunction (42 ischemic and 18 non-ischemic heart disease) underwent ^11^C-HED PET. Myocardial retention was calculated for ^11^C-HED PET as a measure of cardiac sympathetic neuronal integrity. Statistical analysis was performed using Cox proportional hazards regression and log-rank test.

**Results:**

Thirteen deaths (7 cardiac and 6 non-cardiac deaths) occurred during a mean follow-up period of 33 ± 23 months. The patients with death were associated with significantly lower ^11^C-HED retention (7.1 ± 2.1 vs 9.0 ± 2.4, *p* = 0.015) than those without death. The hazard ratio for global ^11^C-HED retention per unit (/min) was 0.762 (*p* = 0.039), which remained significant in multivariate analysis. When the patients were divided into the high (≥8.5) and low (<8.5) ^11^C-HED retention groups, the low ^11^C-HED retention group was associated with significantly poorer survival than the high ^11^C-HED retention group (*p* = 0.004).

**Conclusion:**

The low global ^11^C-HED retention is a marker of poor overall survival in patients with LV dysfunction in this study.

**Electronic supplementary material:**

The online version of this article (doi:10.1007/s12149-016-1081-z) contains supplementary material, which is available to authorized users.

## Introduction

Despite recent advances in therapeutic options, heart failure (HF) continues to be one of the leading causes of mortality and morbidity in many countries [1]. It is well known that autonomic nerve function plays an important role in the pathogenesis and progression of HF [2], which is associated with excessive activation of sympathetic nerve activity [3], and reduction in functional neuronal density [4, 5]. Positron emission tomography (PET) using radio-labeled norepinephrine analogs such as ^11^C-hydroxyephedrine (^11^C-HED) has successfully been used to assess global and regional pre-synaptic sympathetic neuronal integrity of the heart [4, 6, 7]. As compared with more widely available ^123^I- metaiodobenzylguanidine (^123^I-MIBG) imaging [8, 9], it provides better tomographic image quality due to higher counting sensitivity and spatial resolution and the possibility of absolute quantification by routine use of attenuation/scatter correction [10]. However, there are only a few data available focusing on the prognostic value of ^11^C-HED PET [11, 12]; there is none from Japan. Moreover, no ^11^C-HED PET studies have reported all-cause mortality, which is increasingly being utilized as an unbiased endpoint in clinical trials [13].

The aim of this study was to determine whether ^11^C-HED can predict adverse events including all-cause death in Japanese patients with left ventricular (LV) dysfunction.

## Materials and methods

### Study population

This was a retrospective analysis of observational study to characterize HF using imaging biomarkers [10]. We consecutively screened 81 patients who had been referred to The Medical and Pharmacological Research Center Foundation as potential candidates of the study using the following criteria: (1) angiographically proven coronary heart disease (CHD) or non-ischemic symptomatic HF, because both disease conditions are known to cause abnormalities in cardiac sympathetic neuronal integrity, (2) regional or global (left ventricular ejection fraction (LVEF) of <50 %) left ventricular (LV) dysfunction documented by echocardiography, (3) ^11^C-acetate/^11^C-HED PET having been performed under stable general condition within 1 month after the entry, and (4) could be followed-up for >6 months in case of no events. The first criterion was met in all 81 patients; the second criterion in 74 patients; the third and fourth criteria in 60 patients. Patients were excluded if they (1) had unstabilized HF, (2) had acute coronary events (<10 days) such as myocardial infarction or unstable angina prior to the imaging study, or (3) were premenopausal women. There are none who met the exclusion criteria. Thus, 60 patients were finally included in the study. All cardiac medications such as beta-blockers were continued during the study period for safety reasons. After giving a written informed consent in accordance with institutional ethical committee, all patients underwent ^11^C-HED/^11^C-acetate PET imaging.

### Positron emission tomography

**Fig. 1:**
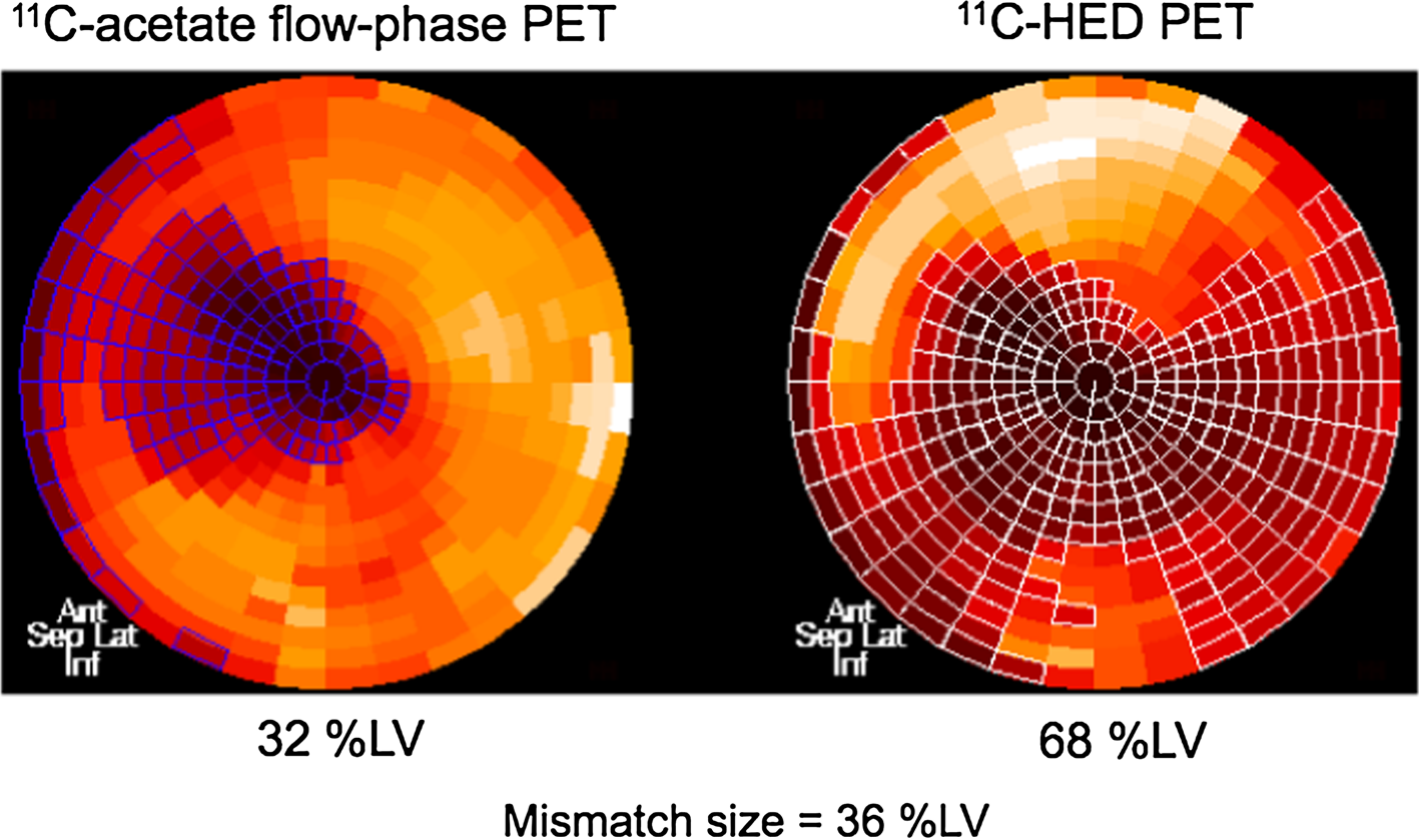
A representative example of ^11^C-acetate perfusion defect size (*left*), ^11^C-HED defect size (*right*), and calculation of mismatch size measurements using polar map analysis. In this example, perfusion defect size, ^11^C-HED defect size, and mismatch size were 32 %LV, 68 %LV, and 36 %LV, respectively

The PET imaging was performed in a manner as described previously [5] using a full-ring PET scanner (Advance, GE Healthcare, Milwaukee, WI, USA). Briefly, transmission scan for 15 min was performed using ^68^Ge/^68^Ga pin sources for attenuation correction. Then, 370 MBq of ^11^C-acetate was intravenously injected, and a dynamic imaging sequence (21 frames, 10 × 10, 1 × 60, 5 × 100, 3 × 80, 2 × 300 s) was acquired for 30 min. In this study, ^11^C-acetate PET data were used to obtain relative perfusion images, which was necessary to measure perfusion defect size and mismatch size as mentioned later. Approximately 120 min after injection of ^11^C-acetate allowing for physical decay of ^11^C, the subjects were re-positioned in the scanner and a second transmission scan was acquired for 15 min. This was followed by intravenous injection of 370–600 MBq of ^11^C-HED, and a subsequent dynamic imaging for 40 min (14 frames, 6 × 30, 2 × 60, 2 × 150, 2 × 300, 2 × 600 s). The total radiation exposure for this imaging procedure was estimated to be <3.4 mSv [14, 15]. Images obtained from a representative case were shown in Fig. 1.

### Echocardiography

Echocardiography was performed in all patients on the same day of PET imaging (between ^11^C-acetate and ^11^C-HED scan) using Vivid 7 (GE Healthcare, Milwaukee, WI, USA) with a 4 MHz transducer. LVEF was measured using Simpson biplane method.

### Processing of PET data

Attenuation-corrected transaxial images were reconstructed using ordered subset expectation maximization algorithm. The image data matrix was 128’’ 128 with pixel size of 2.73 mm and a slice thickness of 4.25 mm. A volumetric sampling procedure was used to create polar maps of relative myocardial perfusion distribution throughout the entire LV myocardium as described elsewhere. In brief, summed data sets of frames 11–13 of the imaging sequence for ^11^C-acetate were used to create polar maps of myocardial activity distribution at 2–4 min after injection, which served as a relative perfusion image.

To obtain global ^11^C-HED retention fraction, the ^11^C-HED uptake averaged for the entire left ventricular myocardium at 30–40 min image was divided by the integral of blood activity curve, which served as a quantitative index of cardiac sympathetic innervation. Then, the polar maps were normalized to the mean of six connected sectors showing the highest overall uptake in the left ventricular myocardium. Defect size on the ^11^C-acetate flow image or ^11^C-HED image was quantified using a cutoff threshold of 60 % of the reference sectors and was expressed as a percentage of the left ventricular myocardium (%LV). Mismatch size was defined as ^11^C-HED defect size minus ^11^C-acetate perfusion defect size. Thus, global ^11^C-HED retention, perfusion defect size, ^11^C-HED defect size, and mismatch size were measured from PET imaging.

### Follow-up

Clinical outcomes were confirmed by patient medical records or telephone interview. The primary endpoint was set to all-cause death; the secondary, cardiac death; and the tertiary, the composite endpoint including HF progression requiring hospitalization, life-threatening arrhythmias such as ventricular tachycardia, and acute coronary syndrome, or death of any cause. In case of multiple events, only the first event was used for analysis.

### Statistical analysis

Data were expressed as mean ± SD unless specified. Statistical analysis was performed using JMP10 (SAS Institute Inc. Cary, NC, USA), or GraphPad Prism6 (GraphPad Software, Inc. San Diego, CA, USA), where appropriate. Wilcoxon rank sum test was used for comparison of variables between the two groups. Proportional difference between the groups was assessed using Chi-square test. Univariate and multivariate analyses using a Cox proportional hazards model were performed. The multivariate model for time to all-cause mortality, cardiac death, or composite endpoint was evaluated using a stepwise forward elimination procedure using variables including sex, age, LVEF, BNP, global ^11^C-HED retention, ^11^C-HED defect size, and mismatch size. In this study, we included only quantitative or objective and discrete variables; those such as NYHA class or presence of hypertension were not included because they were subjective or not necessarily discrete. In addition, the Kaplan–Meier method with log-rank test was applied to obtain survival curves. Optimal cut-off points for continuous variables were determined retrospectively using receiver operating characteristic (ROC) analysis [16]. A *p* value <0.05 was considered significant.

## Results

### Study participants

The clinical characteristics and cause of death are summarized in Table 1 and Supplemantary Table 1, respectively. Of the 42 patients with ischemic etiology, prior myocardial infarction was present in 32 patients. Of a total of 60 patients enrolled, 13 died (7 cardiac and 6 non-cardiac deaths) during the mean follow-up period of 33 ± 23 months (range 1–82, median: 24 months). Of the 7 patients with cardiac death, 2 died of sudden cardiac death and the remaining 5 of progression of HF. Cardiac events occurred in 17 patients, including 13 HF progression, 2 acute coronary syndromes, and 2 life-threatening arrhythmias.

**Table 1:** Clinical characteristics

Age (years)	70 ± 10, 71 (47–89)
Male/female (*n*)	43/17
Etiology	
Ischemic (*n*, %)	42 (70 %)
Prior myocardial infarction (*n*, %)	32 (53 %)
DM (*n*, %)	25 (42 %)
HT (*n*, %)	41 (68 %)
NYHA class 1/2/3/4	15/33/11/1
Medication (*n*, %)	
ACE-I or ARB	34 (57 %)
Aldosterone blocker	16 (27 %)
β-blocker	28 (47 %)
Diuretic	31 (52 %)
Nitrate	39 (65 %)
Ca-antagonist	37 (62 %)

Values are mean ± SD, median (range) or *n* (%)

### All-cause mortality

**Fig. 2:**
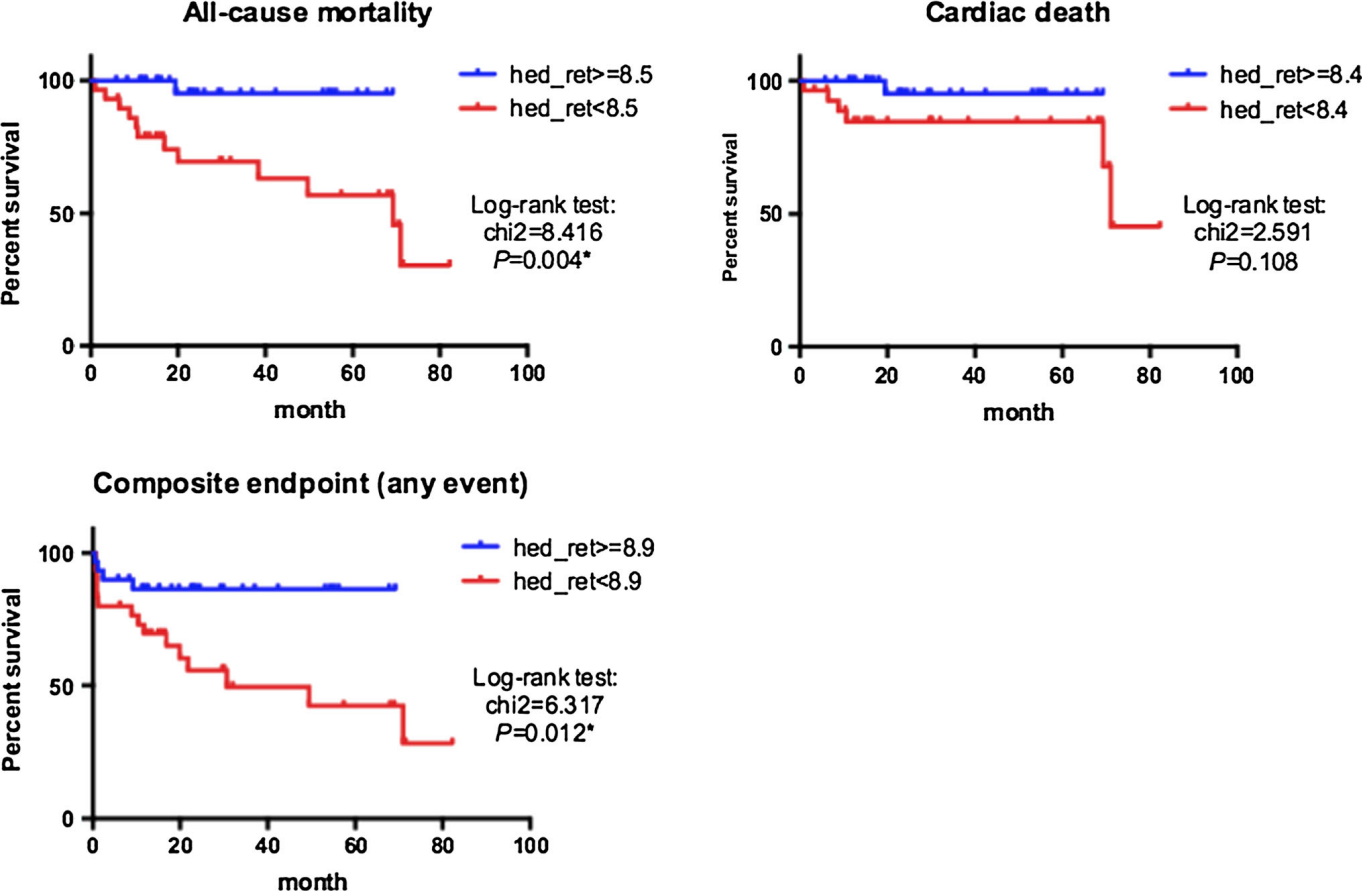
Kaplan–Meier survival curves for all-cause mortality (*upper* *left*), cardiac death (*upper* *right*), and composite endpoint (*lower* *left*) of 2 groups classified by the cut-off value of global ^11^C-HED retention

Imaging parameters, patients characteristics, and serum BNP levels in patients with and without all-cause death are summarized in Table 2. The patients with death were associated with a lower LVEF, lower global ^11^C-HED retention, higher age, and higher serum BNP level than those without death. However, there were no significant differences in sex and perfusion defect size, although there was a trend towards larger ^11^C-HED defect size or mismatch size in patients with death than those without death. The results of univariate and multivariate Cox hazards analysis are summarized in Table 3. The univariate analysis identified age, BNP, and ^11^C-HED retention as a predictor of all-cause death. In the multivariate analysis, age and global ^11^C-HED retention remained significant. When the patients were divided into the high (≥8.5) and low (<8.5) global ^11^C-HED retention groups based on ROC analysis (Supplementary Fig. 1), the low ^11^C-HED retention group was associated with significantly poorer survival than the high ^11^C-HED retention group (*p* = 0.004) (Fig. 2).

**Table 2:** Patients with or without death

	Survived (*n* = 47)	Dead (*n* = 13)	*P* value
Age (years)	69 ± 9	75 ± 12	0.038*
Female (*n*, %)	11 (23 %)	6 (46 %)	0.119
BNP (pg/ml)	176 ± 166	724 ± 1146	0.002*
LVEF (%)	44 ± 13	35 ± 15	0.038*
Perfusion defect size (%LV)	16 ± 17	19 ± 21	0.648
HED retention (/min)	9.0 ± 2.4	7.1 ± 2.1	0.015*
HED defect size (%LV)	30 ± 22	41 ± 26	0.129
Mismatch size (%LV)	14 ± 13	23 ± 20	0.063

Values are mean ± SD or *n* (%)

*BNP* B-type natriuretic peptide, *LVEF* left ventricular ejection fraction, metabolic index, *HED* ^11^C-hydroxyephedrine

* Statistically significant variate (*P* < 0.05)

**Table 3:** Results of univariate and multivariate Cox proportional hazards analysis for all-cause mortality

Variable	Chi-square	HR (CI)	*P* value	Chi-square	HR (CI)	*p* value
Age (per year)	10.86	1.126 (1.047–1.226)	0.001*	12.06	1.135 (1.053–1.243)	0.001*
Female	1.696	2.141 (0.666–6.607)	0.193			
BNP (per 1 pg/mL)	8.620	1.001 (1.000–1.001)	0.003*			
LVEF (per 1 %)	3.098	0.961 (0.916–1.004)	0.078			
Perfusion defect size (per 1 % of LV)	0.018	0.998 (0.964–1.028)	0.895			
HED retetion (per 1/min)	4.274	0.762 (0.580–0.986)	0.039*	5.476	0.759 (0.589–0.957)	0.019*
HED defect size (per 1 % of LV)	1.230	1.013 (0.990–1.038)	0.267			
Mismatch size (per 1 % of LV)	3.351	1.030 (0.998–1.059)	0.067			

* Statistically significant variate (*P* < 0.05)

### Other endpoints

Imaging parameters, patients characteristics, and serum BNP levels in patients with and without cardiac death are summarized in Supplementary Table 2. The patients with death were associated with a higher serum BNP level than those without death. Additionally, there was a trend toward a lower LVEF and larger ^11^C-HED defect size in patients with death than those without death. However, global ^11^C-HED retention did not differ between the 2 groups. Using univariate Cox hazards analysis, LVEF and serum BNP level were significant predictors of cardiac death, of which only serum BNP remained significant in multivariate analysis (Supplemantary Table 3). The Kaplan–Meier analysis showed no significant difference in survival curve between the high (≥8.4) and low (<8.4) global ^11^C-HED retention groups (Fig. 2). When the composite endpoint was applied, the patients with event were associated with a lower LVEF, lower global ^11^C-HED retention, and higher serum BNP level than those without event (Supplementary Table 4). Additionally, there was a trend toward larger ^11^C-HED defect size or mismatch size in patients with event than those without event. Using univariate Cox hazards analysis, LVEF, global ^11^C-HED retention, mismatch size, and serum BNP level were significant predictors of event, of which only serum BNP remained significant in multivariate analysis (Supplementary Table 5). The Kaplan–Meier analysis showed that the low (<8.9) ^11^C-HED retention group was associated with significantly poorer prognosis than the high (≥8.9) ^11^C-HED retention group (*p* = 0.012) (Fig. 2).

## Discussion

The major findings of this study were that (1), of the imaging parameters tested, global ^11^C-HED retention was a significant predictor of all-cause death, whereas global ^11^C-HED retention and mismatch size were predictors of the composite endpoint; (2) in multivariate analysis, age and global ^11^C-HED retention were independent predictors of all-cause death, whereas only serum BNP remained a significant predictor of cardiac death or composite endpoint.

### Sympathetic neuronal imaging and its prognostic value

There is a general consensus that cardiac sympathetic neuronal function plays an important role for the pathogenesis of HF [2]. It is also known that an elevated circulating norepinephrine is a marker of poor outcome [17]. Using imaging techniques with radio-labeled norepinephrine analogs such as ^123^I-MIBG, there are a number of studies showing the prognostic value of cardiac sympathetic neuronal imaging, where the patients with low myocardial ^123^I-MIBG uptake as measured by semi-quantitative heart-to-mediastinum (H/M) uptake ratio are associated with poor prognosis [8, 9, 16, 18–20]. As compared to the aforementioned ^123^I-MIBG imaging, ^11^C-HED PET is considered to be a more sophisticated technique with higher image quality and opportunity of quantification of absolute tracer uptake. Additionally, an advantage of ^11^C-HED PET over ^123^I-MIBG imaging is that it provides higher tomographic image quality and, therefore, is more suitable for regional assessment, as demonstrated in our previous study [10]. However, there are only two studies that have systemically investigated the prognostic value of ^11^C-HED PET due to its limited availability. A retrospective study by Pietila et al. [11] of 46 patients with chronic heart failure (CHF) indicated that CHF patients had significantly lower global ^11^C-HED retention than healthy subjects, and that patients with poor prognosis (death or transplantation) had even lower retention. More recently, Fallavollita et al. [12] have demonstrated that regional myocardial sympathetic denervation assessed by ^11^C-HED PET was predictive for the risk of sudden cardiac arrest in 204 ischemic HF patients who had undergone implantable cardioverter defibrillator therapy. Our results demonstrated that age and low global ^11^C-HED retention were independent predictors of all-cause mortality, which remained true when multivariate analysis was performed, indicating that cardiac sympathetic dysinnervation as measured by ^11^C-HED PET is related to poor survival in patients with LV dysfunction independent of age. Regional parameters such as ^11^C-HED defect size were not significant predictors of all-cause mortality in this study, although there was a trend toward larger perfusion/^11^C-HED mismatch size in patients with death as compared to those without death. This indicates that global rather than regional sympathetic dysinnervation is a better marker of overall mortality. An issue that needs to be addressed is that all-cause mortality includes patients died of non-cardiac disease, including those of malignancy, traffic accident, and unknown cause. Although the exact mechanisms are not clear, it could be possible that the presence of severe HF as reflected by low global ^11^C-HED retention may have unfavorably affected overall survival even in such patients.

Although all-cause mortality is considered to be an objective and unbiased clinical endpoint, the selection of endpoint is still a matter of debate [13]. Therefore, we additionally assessed other endpoints such as cardiac death and composite endpoint. Cardiac death is a cause-specific and commonly used endpoint in clinical studies in patients with heart disease [8, 9, 16, 18–20]. The results showed that, although the patients with cardiac death tended to be associated with a larger ^11^C-HED defect than those without death, the PET derived parameters failed to show predictive power for cardiac death. The small sample size as well as potential misclassification of patients who actually died of cardiac cause as non-cardiac may explain the results. However, serum BNP level, a conventional parameter for HF severity, was still a significant predictor of cardiac death despite the small sample size, indicating that ^11^C-HED parameters have less significant predictive power than serum BNP in this setting. Thus, the prognostic value of ^11^C-HED PET may differ depending on what endpoint is chosen.

For the composite endpoint including any cardiac event and any death, the patients with events were associated with a lower global ^11^C-HED retention and higher serum BNP level than those without event. However, in multivariate analysis, only serum BNP survived as a significant predictor of the endpoint. Whether ^11^C-HED PET parameters would provide incremental prognostic value to serum BNP needs to be addressed in larger trials in future.

A potential advantage of employing ^11^C-HED PET instead of serum BNP would be that regional abnormalities can be assessed using PET technique. As aforementioned, a study by Fallavollita et al. [12] has demonstrated that regional but not global denervation is a predictor of sudden cardiac death. In our study, however, the prognostic value of regional parameters such as HED defect size was not clear mainly because of the limited sample size. Thus, our data have demonstrated that measuring serum BNP is still convenient and useful tool for prediction of cardiac event in any underlying causes.

### Limitations

There are limitations of the study that need to be mentioned. First, the retrospective nature of the study prevented the enrollment of a homogeneous population. Additionally, strict medication control was difficult, where the prevalence of patients with beta-blocker therapy (47 %) was somewhat lower than that (66–96 %) reported in prior imaging studies in HF particularly from western countries [12, 20, 21]. However, this prevalence is not much different from that (54 %) reported in the largest meta-analysis study in Japan [16]. Second, due to the small number of patients, we could not test some cause-specific endpoints such as lethal arrhythmias. Because regional abnormalities in sympathetic innervation is reportedly linked to ventricular arrhythmias [12, 22, 23], this needs to be addressed in further studies. Finally, our patient population consisted of those with ischemic and non-ischemic etiology. In ischemic HF, the presence or absence of prior myocardial infarction may have an impact on prognosis. However, such a specific analysis would require a larger number of patients to draw definitive conclusions. A further study with a large number of ischemic patients is required to address this issue.

## Conclusions

Our results indicate that age and low global ^11^C-HED retention measured by PET are independent markers of poor overall survival in Japanese patients with LV dysfunction in this study. Furthermore, the prognostic value of ^11^C-HED PET may differ depending on what endpoint is chosen.

## Electronic supplementary material

Below is the link to the electronic supplementary material.

Supplementary material 1 (DOCX 113 kb)
