# Supplementary material for: Prediction of all-cause death using 11C-hydroxyephedrine positron emission tomography in Japanese patients with left ventricular dysfunction
Source: Ann Nucl Med. 2016 May 18;30:461–7. doi: 10.1007/s12149-016-1081-z (PMC4961726; doi:10.1007/s12149-016-1081-z)

Supplements

Supplementary Table 1. Events

| Mean follow-up period (months)  All-cause death (n, %)  Classification of death  Cardiac death (n)  Sudden death (n)  Heart failure (n)  Non-cardiac death (n)  Esophageal varix rupture (n)  Pancreas cancer (n)  Traffic accident (n)  Lung cancer (n)  Unknown (n)  Non-fatal cardiac event (n, %)  Classification of event  Lethal arrhythmia  Heart failure progression  ACS | 33 ± 23, 24 (1 – 82)  13 (22 %)  7  2  5  6  1  1  1  1  2  17 (28 %)  2  13  2 |
| --- | --- |

Values are mean ± SD, median (range) or n (%).

Supplementary Table 2. patients with and without cardiac death

|  | without(n = 53) | with (n = 7) | *P*-value |
| --- | --- | --- | --- |
| Age (years)  Female (n, %)  BNP (pg/ml)  LVEF (%)  Perfusion defect size (%LV)  HED retention (/min)  HED defect size (%LV)  Mismatch size (%LV) | 70 ± 10  13 (25 %)  208 ± 264  44 ± 14  16 ± 17  8.8 ± 2.4  31 ± 22  15 ± 13 | 69 ± 13  4 (57 %)  955 ± 1472  32 ± 12  24 ± 20  7.6 ± 2.6  48 ± 26  25 ± 24 | 0.856  0.088  0.001*  0.037*  0.256  0.249  0.057  0.102 |

Values are mean ± SD

* statistically significant variate (*P*<0.05)

Supplementary Table 3. Results of univariate and multivariate Cox proportional hazards analysis for cardiac death

| Variable | Chi-  square | HR  (CI) | *p*-  value | Chi-  square | HR  (CI) | *p*-  value |
| --- | --- | --- | --- | --- | --- | --- |
| Age  (per year) | 0.325 | 1.026  (0.939 – 1.130) | 0.569 |  |  |  |
| Female | 1.641 | 2.751  (0.572 – 14.46) | 0.200 |  |  |  |
| BNP  (per 1pg/mL) | 7.481 | 1.001  (1.000 – 1.002) | 0.006* | 7.481 | 1.001  (1.000 – 1.002) | 0.006* |
| LVEF  (per 1%) | 4.356 | 0.934  (0.867 – 0.996) | 0.037* |  |  |  |
| Perfusion defect size  (per 1% of LV) | 0.300 | 1.012  (0.967 – 1.054) | 0.584 |  |  |  |
| HED retetion  (per 1/min) | 0.594 | 0.871  (0.609 – 1.246) | 0.441 |  |  |  |
| HED defect size  (per 1% of LV) | 2.054 | 1.024  (0.991 – 1.061) | 0.152 |  |  |  |
| Mismatch size  (per 1% of LV) | 2.147 | 1.032  (0.988 – 1.068) | 0.143 |  |  |  |

* statistically significant variate (*P*<0.05)

Supplementary Table 4. patients with and without composite endpoint

|  | without(n = 41) | with (n = 19) | *P*-value |
| --- | --- | --- | --- |
| Age (years)  Female (n, %)  BNP (pg/ml)  LVEF (%)  Perfusion defect size (%LV)  HED retention (/min)  HED defect size (%LV)  Mismatch size (%LV) | 69 ± 9  10 (24 %)  164 ± 152  46 ± 12  15 ± 16  9.1 ± 2.4  29 ± 21  14 ± 12 | 73 ± 12  7 (37 %)  478 ± 970  36 ± 16  19 ± 21  7.7 ± 2.4  40 ± 26  21 ± 19 | 0.149  0.326  0.009*  0.009*  0.486  0.049*  0.096  0.072 |

Values are mean ± SD

* statistically significant variate (*P*<0.05)

Supplementary Table 5. Results of univariate and multivariate Cox proportional hazards analysis for composite endpoint

| Variable | Chi-  square | HR  (CI) | *p*-  value | Chi-  square | HR  (CI) | *p*-  value |
| --- | --- | --- | --- | --- | --- | --- |
| Age  (per year) | 3.123 | 1.044  (0.995 – 1.099) | 0.077 |  |  |  |
| Female | 0.192 | 1.230  (0.461 – 2.960) | 0.661 |  |  |  |
| BNP  (per 1pg/mL) | 14.05 | 1.001  (1.001 – 1.003) | <0.001* | 14.05 | 1.001  (1.001 – 1.003) | <0.001* |
| LVEF  (per 1%) | 6.108 | 0.958  (0.923 – 0.991) | 0.014* |  |  |  |
| Perfusion defect size  (per 1% of LV) | 0.185 | 1.005  (0.980 – 1.028) | 0.668 |  |  |  |
| HED retetion  (per 1/min) | 4.648 | 0.820  (0.685 – 0.982) | 0.031* |  |  |  |
| HED defect size  (per 1% of LV) | 2.453 | 1.015  (0.996 – 1.034) | 0.117 |  |  |  |
| Mismatch size  (per 1% of LV) | 3.874 | 1.027  (1.000 – 1.051) | 0.049* |  |  |  |

* statistically significant variate (*P*<0.05)

Supplementary Figure 1. ROC curves for all-cause mortality (upper left), cardiac death (upper right), and composite endpoint (lower left) by 11C-HED retention.

* statistically significant variate (*P*<0.05)


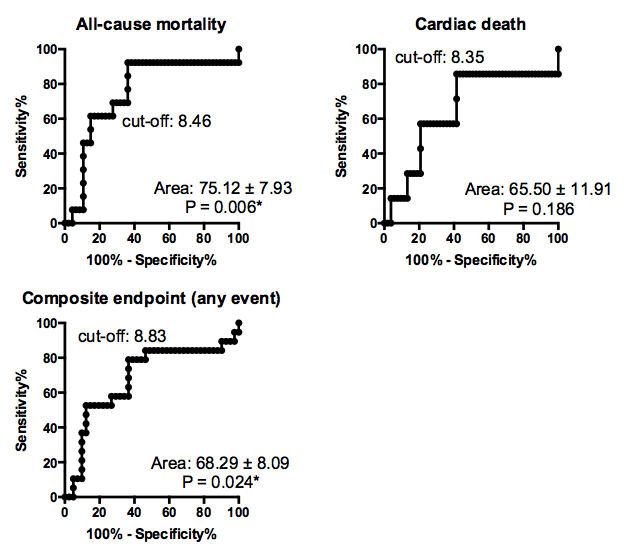

Supplement: Supplementary file 1 — Supplementary material 1 (DOCX 113 kb) [file 12149_2016_1081_MOESM1_ESM.docx]
